# Safety profile of trifluridine/tipiracil monotherapy in clinical practice: results of the German compassionate-use program for patients with metastatic colorectal cancer

**DOI:** 10.1186/s12885-018-5063-5

**Published:** 2018-11-16

**Authors:** Stefan Kasper, Jens Kisro, Martin Fuchs, Christian Müller, Armin Schulz-Abelius, Meinolf Karthaus, Mohammad-Reza Rafiyan, Alexander Stein

**Affiliations:** 1 West German Cancer Center, Department of Medical Oncology, University Hospital Essen, Essen, Hufelandstrasse 55, 45147 Essen, Germany; 2 Community Praxis, Lübeck, Germany; 3 Hospital Bogenhausen, Department of Gastroenterology, Hepatology and Gastrointestinal Oncology, Munich, Germany; 4 Kliniken Essen-Mitte, Department of Medical Oncology, Essen, Germany; 5 Hospital Altenburger Land, Department of Hematology and Oncology, Altenburg, Germany; 6 Staedtisches Klinikum Neuperlach and Harlaching, Department for Hematology and Oncology, Munich, Germany; 7 Department of Hematology and Oncology, Hospital Nordwest, Frankfurt/Main, Germany; 8 University Cancer Center Hamburg, University Medical Center Hamburg-Eppendorf, Hubertus Wald Tumor Center, Hamburg, Germany

**Keywords:** Metastatic colorectal carcinoma, Trifluridine/tipiracil, TAS-102, Real world oncology

## Abstract

**Background:**

Trifluridine/tipiracil (TAS-102, Lonsurf®), a novel oral anti-tumor agent combining an anti-neoplastic thymidine-based nucleoside analogue (trifluridine, FTD) with a thymidine phosphorylase inhibitor (tipiracil hydrochloride, TPI) presents a new treatment option for metastatic colorectal cancer (mCRC) patients refractory or intolerant to standard therapies. FTD/TPI was approved in the European Union (EU) in April 2016 and launched on the German market in August 15, 2016.

**Methods:**

We investigated the characteristics of patients (pts) with mCRC treated with FTD/TPI at 118 centers in Germany from January 12 to August 14, 2016 and analyzed the safety in a clinical real-world setting.

**Results:**

In Germany, a total of 226 mCRC patients were included into a compassionate-use-program (CUP) and received FTD/TPI. For 45.5% of patients (*n* = 101), 253 adverse events (AE) were documented, most of them drug-related (*n* = 135). From January 12 (2016) to March 2 (2017), 124 serious adverse events (SAE) were reported (74 drug related). The most common serious adverse drug reactions (SADR) were leukopenia (12 events), neutropenia (8 events), anemia (7 events), diarrhea and nausea (5 events each) (observation period January 12 2016 to October 7 2016). In total, 122 patients (54%) discontinued FTD/TPI treatment, mostly due to progression (*n* = 75) followed by AEs (*n* = 21), deaths (*n* = 16), and non-specified reasons (*n* = 16). Interestingly, 12 patients with ECOG PS ≥2 achieved up to 3 cycles of FTD/TPI and in this patient population only 3 treatment discontinuations due to AEs were documented and the safety profile was comparable to the entire population.

**Conclusion:**

The patient characteristics as well as the safety profile of FTD/TPI documented in the German CUP were consistent with those reported in the pivotal trial RECOURSE without unexpected safety signals.

## Background

Colorectal cancer (CRC) is the third most common cancer in Germany [1]. During the last two decades, new combination chemotherapies (e.g with oxaliplatin, irinotecan, and flouropyrimidines) and the development of therapeutic monoclonal antibodies (e.g bevacizumab, cetuximab, panitumumab and ramucirumab) and other targeted agents (e.g. aflicercept, regorafenib) have notably prolonged the median survival time of pts. with metastatic CRC (mCRC). In recent clinical trials, the median overall survival (OS) from first-line therapy in metastatic disease has reached approximately 30 months [2–8]. Nevertheless, the prognosis of patients, which are refractory or intolerant to all approved drugs is poor and there is still an unmet medical need for these patients [9–13], especially for those who are in a good performance status and eligible for further therapies.

FTD/TPI is an oral antimetabolite and has been shown to be effective in the treatment of patients refractory or intolerant to approved drugs for mCRC [14]. The drug combines trifluridine, a thymidine-based nucleoside analogue, and tipiracil, which improves the bioavailability of trifluridine by inhibiting the enzyme thymidine phosphorylase, which is involved in its catabolism [15]. In the randomized global phase 3 trial RECOURSE FTD/TPI prolonged OS and progression free survival (PFS) compared to placebo with a favorable safety profile [14]. In the RECOURSE trial, 800 pts. with mCRC refractory or intolerant to fluoropyrimidine, irinotecan, oxaliplation, bevacizumab and to epidermal growth factor (EGFR) antibodies (in pts. with *KRAS* wild-type tumors) were randomly assigned in a 2:1 ratio to either FTD/TPI (35 mg/m^2^ per dose twice daily on days 1 to 5 and 8 to 12, every four weeks) or placebo. In addition in both study arms patients received best supportive care (BSC). FTD/TPI, significantly prolonged median OS compared to placebo (7.1 vs. 5.3 months; hazard ratio (HR) 0.68; *p* < 0.0001). In addition FTD/TPI significantly prolonged median PFS; 2.0 vs. 1.7 Months; HR 0.48; *p* < 0.0001), improved disease control rate ((DCR; 44.0% vs. 16.3%; p < 0.0001) and prolonged median time to deterioration of ECOG performance status compared to placebo. FTD/TPI was well tolerated in the RECOURSE trial. The most overall common adverse events (AE) were leukopenia, neutropenia, anemia and thrombocytopenia and the most common AE ≥ grade 3 was neutropenia (38% of patients treated with FTD/TPI).

However, data from clinical trials does not always reflect clinical experience in a real-life setting. In RECOURSE, for example, patients with ECOG performance status ≥2 were excluded and the rate of patient who had received regorafenib prior to FTD/TPI was only 18%. This is explained by the lack of approval of regorafenib at the time point of initiation of the RECOURSE trial. Thus, little information is available regarding the safety profile of FTD/TPI for patients with poor performance status or patients that had been pretreated with regorafenib.

To collect more information about the usage and tolerability of FTD/TPI in clinical practice, we prospectively investigated the characteristics of patients with mCRC refractory or intolerant to standard chemotherapies treated with FTD/TPI monotherapy within a compassionate use program (CUP), which was conducted from January 12 to August 14, 2016 in designated CRC centers in Germany and compared the rate and severity of AE to the data from the phase III trial RECOURSE. CUPs provide treatment options for patients with unmet medical needs and offer the opportunity to obtain early data on efficacy, safety and use of a new drug in a real-world setting. In our analysis, we differentiated between patients who had received regorafenib prior to treatment with FTD/TPI and those who had not.

## Methods

### Patients and data analysis

This was a prospective study of pretreated patients with mCRC who had received FTD/TPI at 118 designated CRC centers of the German Cancer Society (DKG) with high expertise in treating patients with advanced CRC from 12th January to 14th August 2016. Sites were selected based on the availability of a certification for interdisciplinary management of colorectal cancer by the German Cancer Society (Darmkrebszentrum). All data were collected within the German CUP for FTD/TPI prior to the regular market access in September 2016. All patients must have had a histologically confirmed adenocarcinoma of the colon or rectum and must have been previously treated for metastatic disease with, or have not been candidates for, available therapies including fluoropyrimidines, oxaliplatin, irinotecan, anti-VEGF agents and anti-EGFR agents in case of *RAS* wild-type status. After written informed consent, patients could be included into the CUP if they were ≥ 18 years of age and had adequate organ function and appropriate neutrophil (≥1.5 × 10^9^/l) and platelet (≥75 × 10^9^/l) count. The baseline characteristics collected for each patient included age, sex, ECOG performance status, vital signs (height, weight, body mass index, body surface area and – calculated on the basis of these values - daily dose of FTD/TPI), colorectal surgery (yes/no), *KRAS* or all *RAS* mutational status, time since diagnosis, adjuvant chemotherapy (yes/no), and prior use of regorafenib (yes/no). No data concerning *BRAF* mutational status or mismatch repair deficiency (dMMR) was collected. The body surface area was calculated using the following DuBois formula (all BSA calculations were rounded to 2 decimal places): BSA (m^2^) = ([Body Weight (kg)]^0.425^ x [Height (cm)]^0.725^) × 0.007184 [16]. AE were documented and graded by the investigators using the Common Terminology Criteria for adverse events (CTCAE) in its current version. All data were collected pseudonymously in an electronic data base and were statistically analyzed using the Statistical Analysis System (SAS) version 9.4. software AEs were classified based on the ICH-MeDRA medical terminology.

## Results

### Patient characteristics

A total of 254 patients (58.4% male and 41.6% female) were enrolled into the CUP with FTP/TPI in Germany. Six patients were not eligible for the treatment with FTD/TPI and 22 patients were eligible but did not start treatment. The data of the remaining 226 patients (100%) was included in this analysis. The mean age of patients was 63.15 years with 36.7% of patients being younger than 60 years, 33.2% between 60 and 70 years and 30.1 % older than 70 years, respectively. Of the 226 patients, 28, 62.2, and 9.8% had an ECOG performance status of 0, 1, and ≥ 2, respectively. Median time from diagnosis of metastatic disease until enrolment into the CUP was 36.4 months (5–144 months) with 75.7% of patients having been diagnosed ≥24 months. *KRAS* wild-type tumors were documented for 46.4% of patients, and *KRAS* mutant tumors for 53.6% in our patient population. Comorbidities were documented for 54.4% of patients including hypertension (17.7%), thromboembolic events (15.05%), diabetes mellitus (4.87%), hypothyroidism (4.87%), coronary artery disease (3.1%), polyneuropathy (2.65%), type 2 diabetes mellitus (2.21%), atrial fibrillation (1.77%), benign prostatic hyperplasia (1.77%) and breast cancer (1.77%). Most patients (88.9%) had have colorectal surgery and 36.9% of patients had received an adjuvant chemotherapy. The mean body surface area of the patients was 1.87 m^2^; consequently patients received a mean daily dose of FTD/TPI of 130 mg. In Table 1, patient baseline characteristics of the German CUP-population are summarized.

**Table 1:** Patient characteristics of the German compassionate-use-program (CUP) for FTD/TPIP

Characteristics	Number of Patients in the German FTD/TPI CUP	% of Patients in the German FTD/TPI CUP
Patients (application by medical practitioner)	254	–
Non-eligible patients	6	–
Eligible patients without treatment onset	22	–
Eligible patients with Lonsurf treatment onset	226	100%
Mean age (years)	63.15	
< 60 years	83	36.7%
< 70 years	75	33.2%
> 70 years	68	30.1%
Sex		
Male	132	58.4%
Female	94	41.6%
ECOG performance status^a^		
0	63	28%
1	140	62.2%
≥ 2	22	9.8%
Vital signs		
Mean height (cm)	172.1	–
Mean weight (kg)	74.2	–
Mean body mass index (kg/m^2^) (range)	25 (14.7–43.6)	–
Mean body surface area (m^2^)^c^	1.87	
Mean daily dose of FTD/TPI	130 mg	
Colorectal surgery		
Yes	201	88.9%
No	25	11.1%
KRAS status^a^		
Wild type	103	46.4%
Mutant	119	53.6%
Time from diagnosis of metastatic disease		
< 24 months	55	24.3%
≥24 months	171	75.7%
Prior use of Regorafenib		
Yes	76	33.6%
No	150	66.4%
Adjuvant chemotherapy^d^		
Yes	82	36.9%
No	140	63.1%

^a^4 mCRC patients without KRAS status assessment

^b^This information is missing for 1 patient

^c^The BSA was calculated using the following DuBois formula (all BSA calculations were rounded to 2 decimal places): BSA (m^2^) = ([Body Weight (kg)]^0.425^ x [Height (cm)]^0.725^) × 0.007184

^d^The information for 4 mCRC patients is not available

Abbreviation: not available, n.a

### Pretreatment

Prior enrollment into the CUP and treatment with FTD/TPI, 98.67% of patients received an irinotecan-based regime, 88.05% received an oxaliplatin-based regimen. 97.35% received infusional 5-fluorouracil and 25.66% received capecitabine. The monoclonal VEGF antibody bevacizumab was applied to 85.4% of patients in previous treatment lines, 33.63 and 26.11% had received the monoclonal EGFR antibodies cetuximab and panitumumab, respectively (Table 2). Pretreatment with regorafenib was documented for 33.63%, with aflibercept for 27.88%, and with ramucirumab for 1.77% of patients. Mitomycin was applied to 7.52% of patients before enrollment into the CUP.

**Table 2:** Prior regimes of the mCRC patients in the German compassionate-use-program of FTD/TPI

Lead Substance of Combination Treatment (if applicable)	Number of Patients in the German FTD/TPI CUP	% of Patients in the German FTD/TPI CUP
Fluorouracil	220	97.35%
Irinotecan	223	98.67%
Oxaliplatin	199	88.05%
Bevacizumab	193	85.4%
Cetuximab^a^	76	33.63%
Panitumumab^a^	59	26.11%
Regorafenib	76	33.63%
Aflibercept	63	27.88%
Ramucirumab	4	1.77%
Mitomycin	17	7.52%

^a^some patients received both EGFR antibodies

One mCRC patient each received gemcitabine, carboplatin, pembrolizumab, nintedanib or niclosamide

Abbreviations: *EGFR*, epithelial growth factor receptor; monoclonal antibodies, mabs; not available, n.a

### Treatment

On average, the patients received 2.5 cycles of FTD/TPI within the CUP with a mean dose of 130 mg. FTP/TPI supply within the CUP was stopped in September 2016 after the regular market access was granted. In this context it should be noted that 46% mCRC patients (46%) continued treatment after the regular market access of FTD/TPI. Unfortunately, due to regulatory affairs the follow-up data of these patients were not available. In line, it was not possible to determine the average number of cycles of the whole CUP patient population.

Discontinuation of treatment was documented in 122 patients (54%), in most cases due to progression (61.5%), followed by AEs (17.2%), deaths (13.1%), and non-specified reasons (8.2%). The AEs which lead to discontinuation of FTP/TPI included: general physical health deterioration (4 events), fatigue (3 events), dyspnea (2 events), pyrexia (2 events), anemia, decreased appetite, renal failure, abdominal pain, diarrhea, nausea, leukopenia, decreased weight, constipation, device-related infection and/or hyperbilirubinemia. Interestingly, the time since the last disease progression after the previous treatment line till enrollment into the CUP did not correlate with the likelihood of FTD/TPI treatment discontinuation (Table 3). This suggests that patients with rapid as well as slowly progressive disease benefit from FTP/TPI treatment.

**Table 3:** Last disease progression in the previous treatment line in relation to the discontinuation of treatment with FTD/TPI

Last Disease Progression^a^	Discontinuation of FTD/TPI Treatment				mCRC Patient Number (In Total)
	Yes		No		
	n	%	n	%	n
≤ 7 Days	38	58.72	29	43.28	67
1- < 2 Weeks	25	59.52	17	40.48	42
2- < 3 Weeks	12	52.17	11	47.83	23
3- < 4 Weeks	12	50.0	12	50.00	24
> 4 Weeks	30	47.62	33	52.38	63
					219

^a^Appropriate data from 7 mCRC patients were not available

### Safety

Safety was evaluated for all 226 patients enrolled into the CUP. For 101 patients (45%) 253 AEs were documented, 138 of them reported as drug-related by the investigators (Table 4). The frequency of AEs was comparable to those of the RECOURSE trial, where 524 AEs were reported in 533 patients (Table 4). Most common AEs in the FTD/TPI CUP population were leukopenia (*n* = 25), neutropenia (*n* = 19), anemia (*n* = 15), nausea (*n* = 15), diarrhea (*n* = 11), fatigue (*n* = 9), fever (*n* = 9=, asthenia (*n* = 9) and vomiting (*n* = 7).

**Table 4:** Adverse drug reaction profile for FTD/TPI in German Compassionate-Use-Program (CUP) and the RECOURSE trial

Adverse Events (AE)	FTD/TPI (*n* = 226 patients)			RECOURSE - FTD/TPI^a^ (*n* = 533 patients)	
	Total number	Related	Non-related	Total number Any Grade	Grade ≥ 3
Any adverse event	253	138	115	524	370
Any serious adverse event (SAE) - no. (%)	124^c^ (55)	74^d^ (33)	50^e^ (22)	158 (30)	n.a
Most common AEs^b^ - no. (%)	Total number Any Grade	Serious	Non-serious	Total number Any Grade	Grade ≥ 3
Nausea	15 (5.9)	5 (2.0)	10 (4.0)	258 (48)	10 (2)
Diarrhea	11 (4.3)	5 (2.0)	6 (2.4)	170 (32)	16 (3)
Fatigue	9 (3.6)	5 (2.0)	4 (1.6)	188 (35)	21 (4)
Influenza like illness/Pyrexia/Chills/Fever	9 (3.6)	3 (1.2)	6 (2.4)	99 (19)	7 (1)
General physical health deterioration/Asthenia	8 (3.2)	5 (2.0)	3 (1.2)	97 (18)	18 (3)
Vomiting	7 (2.8)	3 (1.2)	4 (1.6)	148 (28)	11 (2)
Abdominal pain	7 (2.8)	4 (1.6)	3 (1.2)	113 (21)	13 (2)
Decreased appetite	3 (1.2)	1 (< 1)	2 (< 1)	n.a	n.a
Urinary tract infection/Urosepis	3 (1.2)	2 (< 1)	1 (< 1)	n.a	n.a
Constipation/subileus	3 (1.2)	2 (< 1)	1 (< 1)	n.a	n.a
Condition aggravated	3 (1.2)	3 (1.2)	0 (0)	n.a	n.a
Alopecia	2 (< 1)	0 (0)	2 (< 1)	n.a	n.a
Musculoskeletal pain	2 (< 1)	1 (< 1)	1 (< 1)	n.a	n.a
Events associated with fluoropyrimidine treatment					
Stomatitis/Oesophagitis	2 (< 1)	1 (< 1)	1 (< 1)	43 (8)	2 (< 1)
Mucosal inflammation	1 (< 1)	0 (0)	1 (< 1)	n.a	n.a
Oesophageal candidiasis	1 (< 1)	0 (0)	1 (< 1)	n.a	n.a
Hand-foot syndrome	0 (0)	0 (0)	0 (0)	12 (2)	0 (0)
Febrile neutropenia	1 (< 1)	1 (< 1)	0 (0)	20 (4)	20 (4)
Laboratory abnormalities no. /no./total no. (%)					
Leukopenia	25 (9.9)	13 (5.1)	12 (4.7)	407/528 (67)	113/528 (21)
Neutropenia	19 (7.5)	9 (3.6)	10 (4.0)	353/528 (67)	200/528 (38)
Anemia	15 (5.9)	7 (2.8)	8 (3.2)	404/528 (77)	96/528 (18)
Thrombocytopenia	7 (2.8)	4 (1.6)	3 (1.2)	223/528 (42)	27/28 (5)
Increase in total bilirubin	4 (1.6)	3 (1.2)	1 (< 1)	189/526 (36)	45/526 (9)
Renal failure/Increase in creatinine level	2 (< 1)	1 (< 1)	1 (< 1)	71/527 (13)	5/527 (< 1)
Increase in alanine aminotransferase level	0 (0)	0 (0)	0 (0)	126/526 (24)	10/256 (2)
Increase in aspartate aminotransferase level	1 (< 1)	0 (0)	1 (< 1)	155/524 (30)	23/524 (4)
Increase alkaline phosphatase level	1 (< 1)	0 (0)	1 (< 1)	205/526 (39)	42/526 (8)

^a^Data based on Mayer et al., N. Engl. J. Med. 2015; 372: 1909–19

^b^Not all single non-related AEs are listed

^c^SAEs documented for 50 mCRC patients treated with FTD/TPI

^d^Related SAEs documented for 20 mCRC patients treated with FTD/TPI

^e^Non-related SAEs documented for 30 mCRC patients treated with FTD/TPI

Abbreviation: n.a., not available

From 12th January 2016 to 2nd March 2017, 124 (55%) SAEs were reported (74 of them drug related by investigator’s judgment). The incidence of SAEs were slightly higher than in the RECOURSE trial, where 158 (30%) SAEs were reported in 533 patients (Table 4). The most common Serious Adverse Drug Reactions (SADR) in the here reported patient population were leukopenia (13 events), neutropenia (9 events), anemia (7 events), diarrhea, fatigue and nausea (5 events each) during the observation period between 12th of January 2016 to 7th of October 2016. The observed non-hematological SADR were comparable to those reported in the RECOURSE trial (Table 4). However, the incidence of hematological SADRs and other laboratory abnormalities were significantly lower in the German CUP than in the RECOURSE trial.

Notably, 12 patients with ECOG PS ≥2 received treatment with up to 3 cycles of FTD/TPI (4 patients 1 cycle, 6 patients 2 cycles, 2 patients 3 cycles). Among these patients, only 3 discontinuations were due to AEs (cough, fatigue, esophageal candidiasis), the others were due to progression (*n* = 4), death (*n* = 1), and non-specified reasons (*n* = 3). In 9 (40%) out of the 22 patients with ECOG PS ≥2, 24 AEs were reported. The incidence, type and grade of the AEs in patients with ECOG PS ≥2 did not differ from those reported in the entire population of the FTD/TPI CUP (*p* = 0.753, chi-square) (Table 5).

**Table 5:** Adverse event profile for FTD/TPI in mCRC patients with ECOG ≥2

Patient No.^a^	ECOG	MedDRA Preferred Term	Adverse Drug Reaction	
1	2	Death	Non-related	Serious
		Oedema peripheral	Non-related	Non-serious
		Skin ulcer	Non-related	Non-serious
		General physical health deterioration	Non-related	Non-serious
6^b^	2	Cough	Non-related	Non-serious
		Fatigue	Non-related	Serious
		Oesophagitis	Suspected	Serious
37	2	Urinary tract infection	Non-related	Non-serious
45	2	Abdominal Pain	Non-related	Non-serious
		Anaemia	Suspected	Serious
		Constipation	Non-related	Non-serious
		Leukopenia	Suspected	Serious
		Thrombocytopenia	Suspected	Serious
		Urinary tract infection	Suspected	Serious
85	2	Metastases to central nervous system	Non-related	Non-serious
152^b^	3	Dyspnoea	Non-related	Non-serious
210	2	Dyspnoea	Non-related	Non-serious
		Fatigue	Non-related	Serious
		Oedema peripheral	Non-related	Non-serious
		General physical health deterioration	Non-related	Non-serious
230	2	Thrombocytopenia	Suspected	Serious
		Leukopenia	Suspected	Serious
		Nausea	Non-related	Non-serious
248	3	Nausea	Non-related	Non-serious

^a^For 13 mCRC patient (ECOG ≥2) no. 21, 49, 68, 138, 188, 192, 219, 222, 226, 250, 251, 255 and 257 no data of adverse events are available

^b^mCRC patients (ECOG ≥2) with prior Regorafenib treatment

In total 76 patients (69 with ECOG PS 0/1 and 7 with ECOG PS ≥ 2) were pretreated with regorafenib before the enrollment into the CUP. In general, there was no difference in the incidence and type of the AEs or SADRs between patients with and without regorafenib pretreatment (suppl. Table 3). Interestingly, the incidence of diarrhea was slightly higher in regorafenib-pretreated patients.

## Discussion

In our prospective CUP conducted in heavily pretreated patients with mCRC in Germany, we found that patient characteristics as well as the safety profile of FTD/TPI were comparable with those reported in the pivotal RECOURSE trial [14]. Mean age of patients, gender distribution, *KRAS* mutational status of patients as well as time from diagnosis of colorectal cancer was consistent between the German CUP and the phase 3 trial, though the mean body surface area in the CUP was slightly higher (1.87 vs. 1.781, respectively) leading to a higher daily dose of FTD/TPI (130 mg) compared with the RECOURSE trial (120 mg) (suppl. Table 1). However, in comparison with the RECOURSE trial, the patient population within the German CUP had two major differences indicating that phase 3 trials do not always reflect clinical experience in a real-life setting. First, in our study 9.8% of patients with an ECOG performance status of ≥2 were included whereas patients with poor performance status were excluded in the RECOURSE trial (0%). Thus, our cohort represents a more “real” patient population in daily clinical practice. We do not observe any remarkable difference in the safety profile of FTD/TPI or in the duration of treatment in patients with an ECOG PS ≥2. Among the 22 patients with ECOG PS ≥2 only 3 discontinued treatment with FTD/TPI due to AEs. Thus FTD/TPI seems to be tolerated and effective even in patients with poorer performance status. We did not detect any unexpected safety signals with comparable characteristics and incidence of AEs and SAEs. However, the incidence of hematological AEs and other laboratory abnormalities was lower compared to the RECOURSE trial, presumably due to a underreporting of these AEs in CUPs. This is in line with the findings of a postmarketing surveillance study in 3.420 patients treated with FTD/TPI from May to November 2014 in Japan [17]. In this large observational study the safety profile was also similar to those from the RECOURSE trial. Furthermore, an expanded-access program (EAP) in the USA was carried out to further assess the safety profile of FTD/TPI in a real-world setting in 549 US patients with refractory mCRC. In this EAP, patients had a comparable exposure duration to that reported in 64 US patients who had participated in the RECOURSE trial, with no unexpected safety concerns [18]. In the EAP, only 4% of patients discontinued the treatment with FTD/TPI due to AEs [18]. Finally, a recent Spanish CUP conducted with refractory 538 mCRC patients supported the findings of the German CUP presented here. In the Spanish CUP, FTD/TPI was generally well tolerated and the real-world data analysis was consistent with that reported in phase 3 trials of FTD/TPI in patients with mCRC [19]. In contrast to other European countries regorafenib is not longer available for patients in Germany since 2016. For this reason the number of patients in the German CUP, which were pretreated with regorafenib, is significantly lower than in patients of other European CUPs published recently. For example 70% of patients in the Italian CUP were pretreated with regorafenib compared to only 33.6% in the here reported patient population [20]. In addition, in Germany patients with advanced CRC are not only treated in large academic centers but also in smaller hospitals or private practices. For this reason 118 centers participated in the German CUP and enrolled 226 patients in total. So, there was a high variability in the experience of treating advanced CRC among the physicians. In contrast in other European countries like the Netherland the treatment of patients with advanced cancers is more centralized. In line, in the Netherlands’ CUP 148 patients were enrolled in only 17 centers [21]. Taken together, the here reported patient population of the German CUP differs from those previously reported.

The data from this CUP is limited because long-term follow-up to analyze survival outcomes of the patients was not allowed due to regulatory affairs. Nevertheless, our study confirms findings of previous data that the safety profile of FTD/TPI was comparable to the pivotal trial RECOURSE and that the drug is well tolerated in a real life setting [18, 19, 22]. Moreover our data show that patients with an ECOG performance status of ≥2 and/or previously treated with regorafenib can recieve FTD/TPI safely and effectively. Further clinical trials should investigate these patient groups with respect to efficacy as well as safety with a focus on the sequence of FTD/TPI and regorafenib.

## Conclusion

In a prospective CUP in Germany conducted with pretreated mCRC patients the safety profile of FTD/TPI was investigated in a real-world setting. FTP/TPI was well tolerated in clinical practice. The patient characteristics and the toxicities of FTD/TPI documented in the German CUP were comparable with those reported in the pivotal RECOURSE trial without any unexpected safety signals. However, in the German CUP a higher number of patients had an ECOG performance status of ≥2 (9.7% vs. 0% in RECOURSE) and the previous use of regorafenib was more common in the real-life setting (33.6% vs.18% in RECOURSE).

### Clinical practice points

- The findings of this German CUP in line with other European, Asian and US American CUPs show that the safety profile of FTD/TPI in a real-world setting is comparable to that reported in the RECOURSE trial.
- FTD/TPI is generally well tolerated in clinical practice, even in patients with poorer performance status.
- In Germany, FTD/TPI is currently the only effective and safe anti-tumor agent available for patients with refractory mCRC.
